# *Smilax china* root extract as a novel Glucose- 6-phosphate dehydrogenase inhibitor for the treatment of hepatocellular carcinoma

**DOI:** 10.1016/j.sjbs.2022.103400

**Published:** 2022-07-30

**Authors:** Lubna Kanwal, Shaukat Ali, Azhar Rasul, Hafiz Muhammad Tahir

**Affiliations:** a Department of Zoology, University of Okara, Okara, Pakistan; b Applied Entomology and Medical Toxicology Laboratory Department of Zoology, Government College University Lahore, Pakistan; c Department of Zoology, Government College University Faisalabad, Pakistan

**Keywords:** *Smilax china*, Hepatocellular carcinoma, Cancer cell metabolism, Glucose- 6- phosphate dehydrogenase, G6PD, Glucose-6-phosphate dehydrogenase, HCC, Hepatocellular carcinoma, PPP, Pentose phosphate pathway, NADPH, Nicotinamide adenine dinucleotide phosphate, NBT, Nitroblue tetrazolium, PMS, Phenazine methosulphate

## Abstract

A novel therapeutic strategy for cancer treatment is to target altered tumor metabolism. Glucose- 6-phosphate dehydrogenase (G6PD) has been recently discovered to be implicated in apoptosis and angiogenesis, making it an excellent target in cancer treatment. The current study aimed to screen the plant extracts library to find potent hits against G6PD through enzymatic assay. Protein expression was induced by IPTG and purified using Ni-NTA columns after transformation of the pET-24a-HmG6PD plasmid into *E. coli* BL21-DE3 strain. An enzymatic assay was established by using purified rG6PD protein, for the screening of G6PD inhibitors. Out of 46 plant extracts screened, the sixteen plant extracts have shown inhibitory activity against the G6PD enzyme. At doses from 1 to 4 µg/ml, this extract demonstrated concentration-dependent inhibition of G6PD with an IC_50_ value of I.397 µg/ml. Moreover, the anticancer activity evaluation against HepG2 cells determined *Smilax china* as a potent inhibitor of cancer cells (IC_50_ value of 16.017 μg/ml). The acute and subacute toxicities were not observed in mice with various concentrations (50, 100, 200 and 2000 mg/kg). Furthermore, to identify the compounds from *Smilax china* as G6PD inhibitors, a literature-based phytochemical investigation of *Smilax china* was conducted, and sixty compounds were docked against the NADP+ and G6P binding sites of G6PD. The results of this study showed that three compounds were Scirpusin A, Smilachinin and Daucosterol with MolDock Score of −156.832, −148.215, and −145.733 respectively, against NADP+ binding site of G6PD. Conclusively, *Smilax china* root extract could be a safer drug candidate for the treatment of hepatocellular carcinoma.

## 1 Introduction

Cancer is a multifaceted disease that has multiple transformations that arise at the metabolic, epigenomic, genomic, transcriptomic and proteomic levels (Missiroli et al., 2020). Cancer is not simply restricted to unusual cell multiplication; rather it shows heterogeneity regarding a few basic cell changes, such as self-control of growth signals, passiveness to anti-growth signals, evasiveness of cell death, immense replicative capability, consistent metastasis and vasculature (Hanahan and Weinberg, 2000). Cancer is a non-communicable disease with maximum deaths around the globe. One of the main reasons for cancer-related demises around the globe is liver cancer. Due to the increased prevalence of hepatitis B or C infections, alcohol intake, nonalcoholic fatty liver disease, and other variables, its prevalence rate has progressively increased in the last decade (Lin et al., 2017).

For growth and survival, cancer cells have a changed metabolism, anaerobically metabolizing glucose, with enhanced lactate generation, as observed by Otto Warburg in 1924 (Missiroli et al., 2020). Over the last two decades, a significant consideration has been made to cancer metabolism, particularly in terms of glucose metabolism (Vazquez et al., 2016). The pentose phosphate pathway (PPP) is the main part of glucose metabolism (Yang et al., 2019). Altered metabolism is thought to be a key feature of tumorigenesis since it can regulate important activities including invasion, migration and proliferation (Missiroli et al., 2020). Tumor cells significantly depend on various metabolic pathways to assist their metastasis, advancement and viability (Alannan et al., 2020).

Recently, the PPP has been discovered to contribute significantly to malignant cell development by equipping with nucleotide precursors, which are required for cell multiplication, as well as nicotinamide adenine dinucleotide phosphate (NADPH), which is used not only for intracellular Reactive oxygen species (ROS) detoxification but catabolic metabolism also (Mele et al., 2018). Although less efficient in terms of ATP synthesis, cancer cells increased reliance on aerobic glycolysis fulfills not only the energy demands of rapidly growing cancer cells but also the higher demands for metabolic intermediates required for anabolic processes (Varghese et al., 2020). The hexokinase enzyme changes glucose to glucose-6-phosphate, which can subsequently be metabolized further by glycolysis or the PPP, as glucose is transported through glucose transporters in the cell. As a result of the enormous biosynthetic requirements of quickly developing cancer cells and their need to adjust to distressing conditions, the PPP has been recommended to advance cancer development and treatment refusal. Subsequently, a large number of the enzymes that make up the PPP, including G6PD are related to malignancy (Hu et al., 2015). The pentose phosphate pathway's initial step is catalyzed by G6PD, which is important for NADPH production. G6PD is commonly considered the pioneer enzyme to generate NADPH in tumor cells after the PPP is initiated (Ju et al., 2017, Pinna et al., 2019).

G6PD is linked to the control of cell growth and transformation as the basic rate-limiting enzyme in the PPP (Pu et al., 2015). The enzyme G6PD is linked with a poorer patient diagnosis and is overexpressed in human cancers (He et al., 2018). The discovery that G6PD plays a key role in tumor cell metabolism has invigorated researchers to look for ways to specifically limit G6PD activity in cancer patients (Pes et al., 2019). Consequently, this current research was focused to assess the capability of different extracts of plants from Pakistani flora against G6PD. As a result plant extracts library was screened using an in vitro enzymatic assay technique to identify G6PD inhibitors. Based on biochemical and cell-based findings presented here, *Smilax china* root extract targets G6PD and has an anti-cancer effect against HepG2 cells.

## 2 Materials and methods

### 2.1 Ethical statement

Animal trials were conducted in accord with local (law of Government College University, Lahore, Pakistan) and international law (Wet op de dierproeven, Wod, Article 9 of Dutch Law as mentioned in our previous reports (Dar et al., 2019, Mughal et al., 2019, Mumtaz et al., 2019, Ali et al., 2020, Mughal et al., 2020a, Mughal et al., 2020b).

### 2.2 Plant extract library

The established library of plant extracts at GCUF (Rasul et al., 2021) was screened for the identification of potent hits against G6PD.

### 2.3 Expression and purification of rG6PD protein

Plasmid for G6PD was presented by Prof. Dr. Katja Becker, a German Physician and Biochemist. The G6PD sequence was cloned into the pET24a expression vector (Novagen) and overexpressed in *E. coli* BL21 cells (Invitrogen) carrying pRAREII with a His tag at the C-terminal. At 23 °C, overexpression was carried out in 2xYT medium (16 g. tryptone, 10 g yeast, 5 g NaCl per liter medium) added with chloramphenicol (12.5 µg/ml) and kanamycin (50 µg/ml). IPTG (0.1 mM) was used to induce protein expression at a 600 nm optical density. The collection, lyses, and purification of cells was done using Ni-NTA metal affinity chromatography after 24 h. For lyses and purification of G6PD, 50 mM Tris/HCl, 300 mM NaCl, 0.1 mM NADP^+^, pH 8.0 was used as a buffer. From the Ni-NTA column, G6PD was eluted using 150 and 300 mM imidazole and kept at 4 °C with 1.8 M ammonium sulfate +0.1 mM NADP^+^ (Preuss et al., 2013).

### 2.4 Conduction of G6PD enzymatic assay

Purified G6PD was tested for inhibitors using an enzymatic activity assay. The G6PD activity was detected by a rise in optical density at 340 nm because of NADPH production. The use of a multi-well plate to measure absorbance at 340 nm is incompatible. Consequently, for G6PD activity a new colorimetric was developed (Zara et al., 2022).

### 2.5 HepG2 cell culture

The HepG2 cell line of human liver cancer was grown in Dulbecco's Modified Eagle Medium (DMEM) added with 10 % FBS and 100 IU/mL penicillin streptomycin. HepG2 cells were kept in a CO_2_ incubator and allowed to grow at 37 °C with 5 % CO_2_ supply (Rasul et al., 2021).

### 2.6 MTT cytotoxicity assay

The anti-cancer properties of extracts of plants were evaluated by MTT assay. HepG2 cells were grown on 96-well plates for this experiment. The malignant cells were given varying concentrations of the extract after an overnight incubation period. Then cells were incubated for 4 h at 37 °C with 20 μg/ml of MTT solution. Following this, media was aspirated with the addition of 100 µL of DMSO. Finally, using an ELISA plate reader (Thermo Scientific), absorbance was observed at 540 nm wavelength (Zara et al., 2022).

### 2.7 Toxicity profiling

Toxicity profiling was done to identify the adverse effects of *Smilax china* root extract. Swiss albino mice, male and female, weighing 20–30 g and aged 5–6 weeks, were provided by the Department of Zoology, Government College University, Lahore, Pakistan. The mice were kept in conventional circumstances (23–25 °C, 12 h/12 h light/dark cycle) with free access to a standard pelleted feed and water *ad libitum*. Before beginning the trial, they were given a week to acclimate to laboratory settings. The mice were split into two groups: one for acute and one for subacute toxicity testing. The sub-acute toxicity study group was further divided into four groups with 5 mice each. The animals were weighed once a week, and morphological and behavioral changes, as well as food and water consumption, were all observed. In the acute toxicity group, the mice were kept for 72 h after receiving a single dose of 2000 mg/kg orally, and subsequently sacrificed. Mice in the sub-acute toxicity group were dissected after 28 days of treatment, and blood specimens and organs were obtained for analysis. The kidney and liver tissues were removed and examined for any abrasion. In both the treatment and control groups, individual organs were weighed and their features were correlated.

### 2.8 Histopathological assessment

The kidney and liver of mice were removed and kept in 10 % formalin. After dehydrating the tissues in alcohol, they were fixed in paraffin and sliced into 4–5 µm slices. Hematoxylin and eosin staining were applied to examine the tissue slices under a photomicroscope at various magnifications (Ishtiaq et al., 2017).

### 2.9 Docking studies

To assess the probable binding locations and affinity of chemicals discovered in the extract, a molecular docking study was conducted. The human G6PD X-ray crystallographic structure was derived from the https://www.rcsb.org/structure/2BHL. Proteins were prepared for docking by importing them into Molegro Virtual Docker (Molegro. 2011). Water molecules were removed from the crystal structure, and protein structural defects were evaluated. Docking was used to determine the binding sites for glucose 6-phosphate (G6P) and nicotinamide adenine dinucleotide phosphate (NADP+). MolDockScore was used to report the results. By selecting the reference ligand center, each docking pit was given 16 Å radiuses. Discovery Studio Visualizer 2021software was used to analyze the binding poses. The phytochemicals were found using PubChem, and their 3D SDF Conformers were retrieved using InChI Key Codes from the ZNC database. The UCSF Chimera Software was used to prepare them for docking.

## 3 Results

### 3.1 Expression and purification of rG6PD protein

**Fig. 1:**
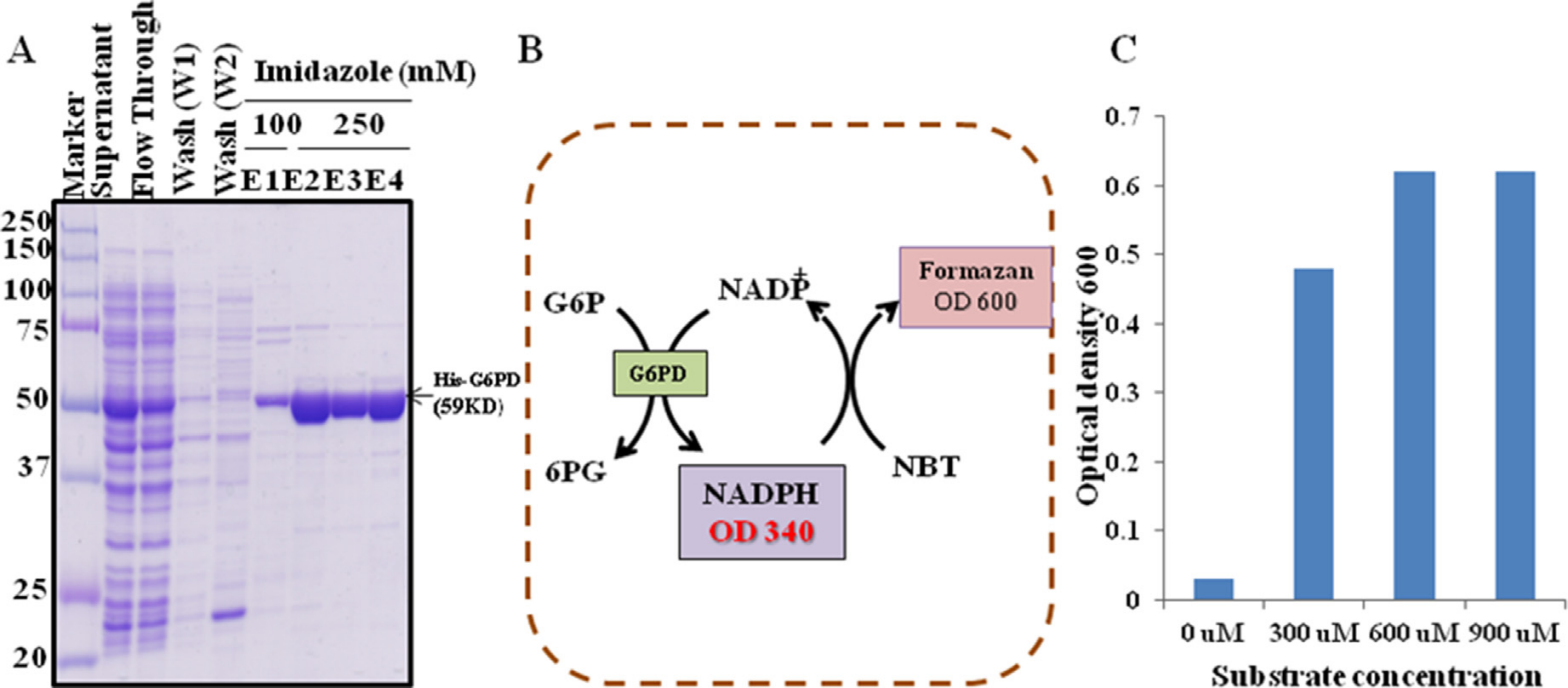
(A) Purity test of the purified rG6PD protein; (B) Principle of G6PD enzymatic activity assay; (C) Development of substrate concentration (G6P) for G6PD enzymatic assay.

In BL21-DE3 *E. coli* cells, a recombinant 6his-G6PD plasmid was expressed. The recombinant C-terminal his-tagged protein was purified from *E. coli* cells using Ni-NTA affinity chromatography. SDS-PAGE was used to evaluate the purified recombinant protein. An approximate 59 kDa band on SDS PAGE shows the effective expression of G6PD recombinant protein in BL21-DE3 *E. coli* clones (Fig. 1A and 1B).

### 3.2 Validation and establishment of G6PD enzymatic assay

G6PD enzymatic assay was developed using purified rG6PD protein, based on the principle that G6PD activates the G6P to convert into 6-phosphoglucono-δ-lactone and produces NADPH. The NADPH that is produced when G6P interacts with its substrate reacts with nitroblue tetrazolium (NBT) and phenazine methosulfate (PMS) to produce formazan. At 600 nm, the absorbance of formazan was measured according to this assay. The enzymatic activity of G6PD was monitored spectrophotometrically by measuring the decreased NADH at 340 nm. Various concentrations of protein and substrate were used to optimize reaction conditions. The enzymatic activity of G6PD was measured at varying G6P concentrations (Fig. 1C). Based on our findings, a substrate concentration of 600 µM was selected for further testing.

### 3.3 Screening a library of crude plant extracts by in vitro G6PD enzymatic assay

The inhibitory capacity of 46 extracts derived from various sections of 34 plants spanning over 20 families of Pakistani flora against G6PD was determined using an established coupled G6PD enzymatic assay. These extracts were tested at 400 μg/ml in this preliminary screening, and the obtained results are shown in Table 1. Out of 16 plant extracts that were found active against G6PD, 11 extracts exhibited the highest inhibitory activity, and three extracts were found to be moderately active, whereas two plant extracts were slightly active against G6PD.

**Table 1:** Preliminary screening of a library of crude plant extracts to identify G6PD inhibitors.

Sr. No.	Family	Plant name	Common name	Part used	G6PD Activity
1	Fabaceae	*Dalbergia sissoo*	Indian rosewood	Seeds	**–**
				Bark	**–**
		*Cyamopsis tetragonoloba*	Guar gum	Seeds	**–**
		*Albizia lebbeck*	Lebbeck	Flowers	**–**
				Seeds	**–**
				Seed coat	**–**
				Leaves	**–**
		*Cassia fistula*	Golden shower	Leaves	+ +
				Fruit	**–**
		*Cicer arietinum*	Chickpea (White)	Seeds	**–**
			Chickpea (Black)	Seeds	**–**
		*Trigonella foenumgraecum*	Fenugreek	Seeds	**–**
		*Acacia farnesiana*	Thorn mimosa	Seeds	**–**
2	Apocynaceae	*Calotropis procera*	Sodom apple	Leaves	+ +
		*Nerium oleander*	Oleander	Leaves	**–**
3	Meliaceae	*Azadirachta indica*	Indian lilac	Leaves	+ + +
4	Asteraceae	*Artemisia absinthium*	Common wormwood	Whole plant	**–**
		*Helianthus annuus*	Sunflower	Seeds	+ + +
		*Ageratum conyzoides*	Goat weed	Whole plant	**+**
5	Cucurbitaceae	*Momordica charantia*	Bitter melon	Vegetable	**–**
				Seeds	**–**
		*Cucumis melo agrestis*	Wild melon	Leaves	**–**
				Stem	**–**
		*Citrullus colocynthis*	Desert bitter gourd	Fruit	**–**
6	Oxalidaceae	*Oxalis corniculata*	Creeping woodsorel	Whole plant	**–**
7	Asphodelaceae	*Aloe barbadensis*	Aloe vera	Whole plant	+ + +
		*Asphodelus tenuifolius*	Wild onion	Whole plant	**+**
8	Malvaceae	*Bombax ceiba*	Cotton tree	Leaves	**–**
				Bark	+ + +
9	Amaranthaceae	*Chenopodium album*	Lamb's quarters	Whole plant	**–**
10	Smilacaceae	*Smilax china*	China root	Roots	+ + +
11	Myrtaceae	*Eucalyptus camaldulensis*	Himalayan poplar	Bark	+ + +
12	Sapindaceae	*Litchi chinensis*	Lychee	Seeds	+ + +
				Bark	+ + +
				Leaves	+ + +
13	Lythraceae	*Lowsonia inermis*	Henna	Leaves	+ +
		*Punica granatum*	Pomegranate	Seed coat	+ + +
				Seeds	**–**
14	Cyperaceae	*Cyperus esculentus*	Watergrass	Flowers	+ + +
15	Zygophyllaceae	*Fagonia arabica Linn*	Dhamasa	Whole plant	**–**
16	Solanaceae	*Solanum nigrum*	Black nightshade	Whole plant	**–**
17	Apiaceae	*Trachyspermum ammi*	Carom seeds	Seeds	**–**
		*Coriandrum sativum*	Coriander	Seeds	**–**
18	Umbelliferae	*Ferula asafetida*	Heng	Resin	**–**
19	Linaceae	*Linum usitatissimum*	Flax seeds	Seeds	**–**
20	Rutaceae	*Citrus maxima*	Chinese grapefruit	Peel	**–**

+++ = > 80 % inhibition; ++ = 60–80 % inhibition; + = < 60 %, inhibition; ─ = 0 % inhibition.

**Fig. 2:**
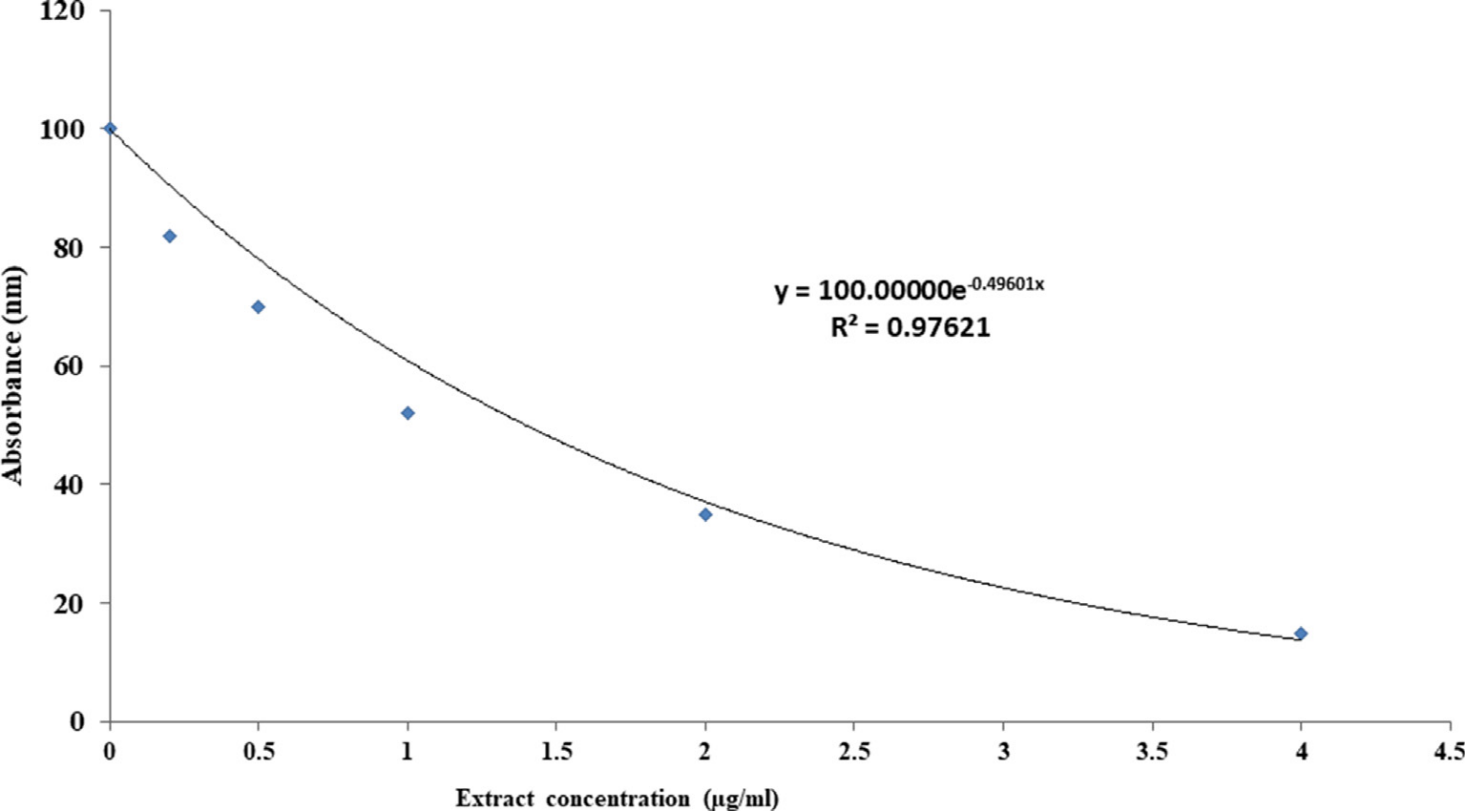
The dose–response curve showing the decrease in absorbance after treatment with 0, 1, 2, 3, 4 μg/ml of *Smilax china* root extract.

Screening of hits was continued at decreasing doses to discover the most effective plant extracts at smaller concentrations. Out of these highly active plant extracts, *Smilax china* root extract was tested dose-dependently at various concentrations (1–4 μg/ml) in the re-confirmation assay and the dose–response curve was obtained (Fig. 2). The findings suggest that root extract of *Smilax china* could be used to further identify and isolate potential compounds for G6PD inhibition. Thus, this plant extract was selected for additional cytotoxicity testing against Hepatocellular carcinoma.

### 3.4 Evaluation of cytotoxicity of *Smilax china* root extract against HepG2 cells and calculation of IC_50_ value

**Fig. 3:**
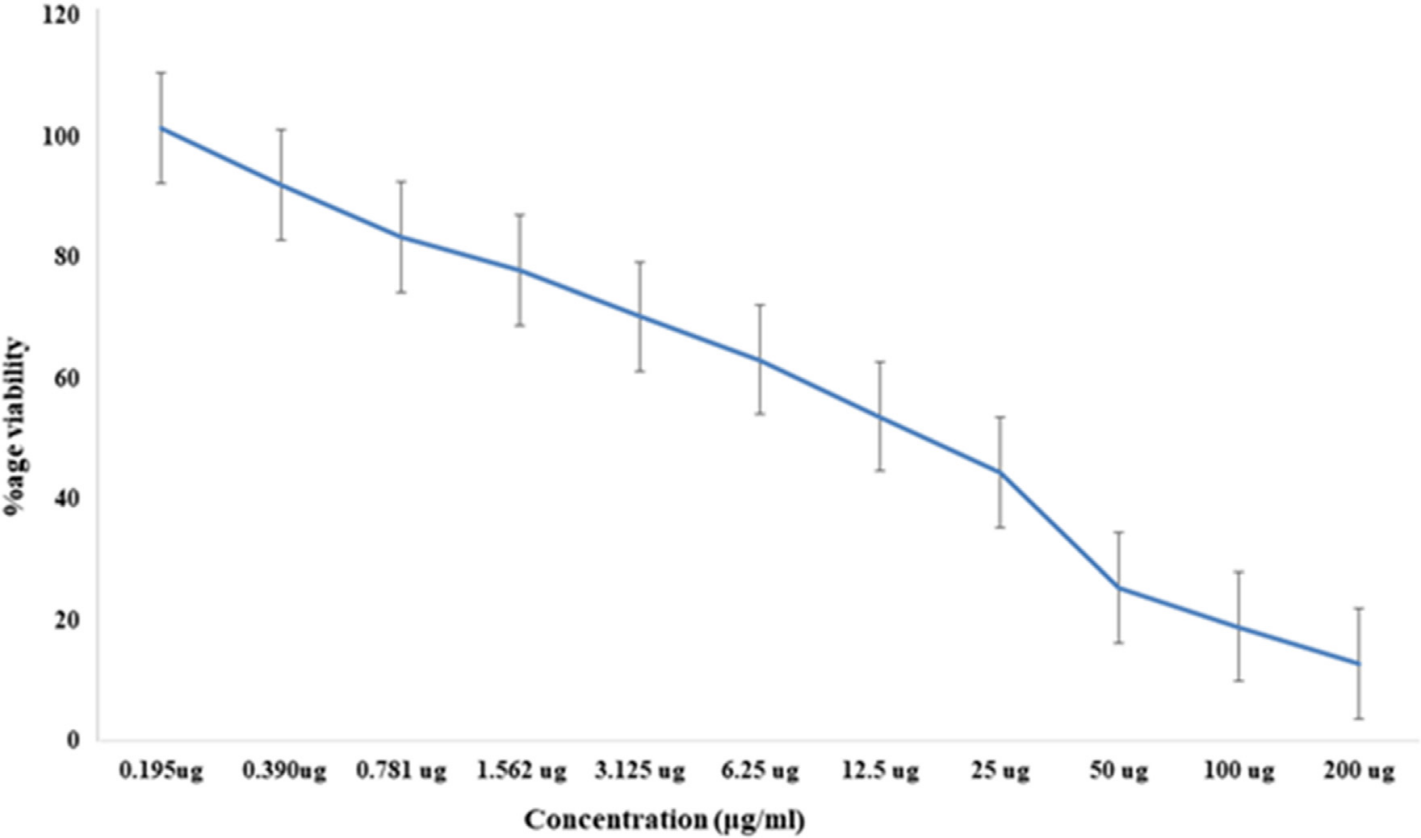
*Smilax china* root extract inhibited the growth of HepG2 cells with an IC_50_ value of 16.017 μg/ml. Cancer cells were treated with 0.195, 0.390, 0.781, 1.562, 3.125, 6.25, 12.5, 25, 50, 100 and 200 μg/ml of *S. china* root extract for 24 h.

HepG2 cell line was given different doses of *Smilax china* root extract (0.195, 0.390, 0.781, 1.562, 3.125, 6.25, 12.5, 25, 50, 100 and 200 μg/ml). To calculate the IC_50_ value, dose–response curves were created. *Smilax china* root extract has the potential to repress the proliferation of HepG2 cells with an IC_50_ of 16.017 μg/ml (Fig. 3).

### 3.5 Acute toxicity study

All animals were kept under observation and carefully monitored for general behavior and development of any lethal signs or symptoms for 72 h. All mice survived for up to 72 h after taking a single dosage of 2000 mg/kg body weight of methanol root extract via oral administration. All the animals in the extract-treated group were normal, with no apparent difference in behavior, food and water consumption and body weight (Table 2).

**Table 2:** Clinical signs and general behaviors observed during acute toxicity study.

Sr. No.	Parameters	Swiss Albino mice treated at a single dose of 2000 mg/kg				
		1	2	3	4	5
1	Convulsions	Not found	Not found	Not found	Not found	Not found
2	Salivation	Not found	Not found	Not found	Not found	Not found
3	Lethargy	Not found	Not found	Not found	Not found	Not found
4	Lacrimation	Not found	Not found	Not found	Not found	Not found
5	Drowsiness	Not found	Not found	Not found	Not found	Not found
6	Nasal bleeding	Not found	Not found	Not found	Not found	Not found
7	Food consumption	Normal	Normal	Normal	Normal	Normal
8	Water consumption	Normal	Normal	Normal	Normal	Normal
9	Body weight	27.79 g	27.45 g	22.13 g	31.22 g	25.87
10	Mortality	Not found	Not found	Not found	Not found	Not found

### 3.6 Sub-acute toxicity study

All animals were treated with repeated oral doses of the extract (50, 100, or 200 mg/kg) in the sub-acute toxicity study and showed no signs of toxicity or mortality. At the end of the trial and for the entire 28-day period, both the control and treated mice appeared healthy and active.

### 3.7 Organ and body weight assessment

The animals' body weights and the weights of their vital organs such as liver and kidneys were calculated and listed in Table 3. In the subacute toxicity trial, all animals in the treatment groups (50, 100 and200 mg/kg) gained weight normally in comparison to the control group. The body weights gradually increased from day one to the end of the trial in all experimental groups related to their body weights at the start of the study, however, the maximum rise was recorded in the mice given 200 mg/kg of the extract. The results revealed that the liver and kidney were not adversely affected throughout the treatment; however, there was a noticeable rise the in weight of the liver of mice in the acute toxicity study group.

**Table 3:** Effect of *Smilax china* methanolic extracts acute and sub-acute oral administration on organs and body weight in Swiss albino mice.

Groups	Treatment (mg/kg/bw)	Initial body weight (g)	Final body weight (g)	Vital organs weight(g)	
				Liver	Kidney
**Subacute toxicity**					
I	Control	30	28.103	1.533	0.370
II	50	24.4	26.954	1.436	0.3
III	100	21.8	24.47	1.46	0.246
IV	200	20	29.122	1.534	0.342
**Acute toxicity**					
V	2000	27.4	26.89	1.608	0.325

### 3.8 Histopathological examination of organs

**Fig. 4:**
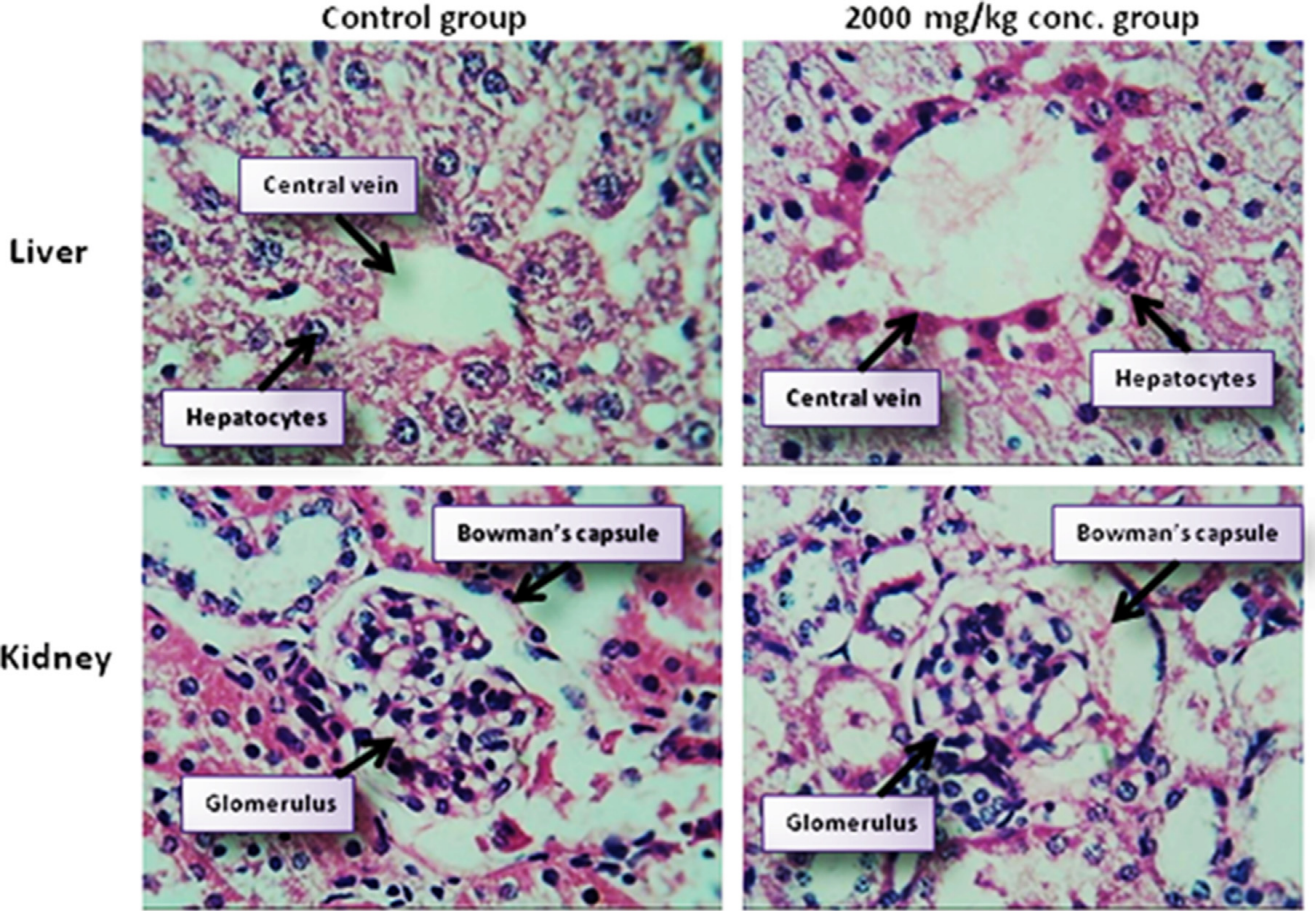
Histopathological examination of Liver and Kidney tissues of Swiss Albino mice at 40X after a single dose of 2000 mg/kg in acute toxicity study.

**Fig. 5:**
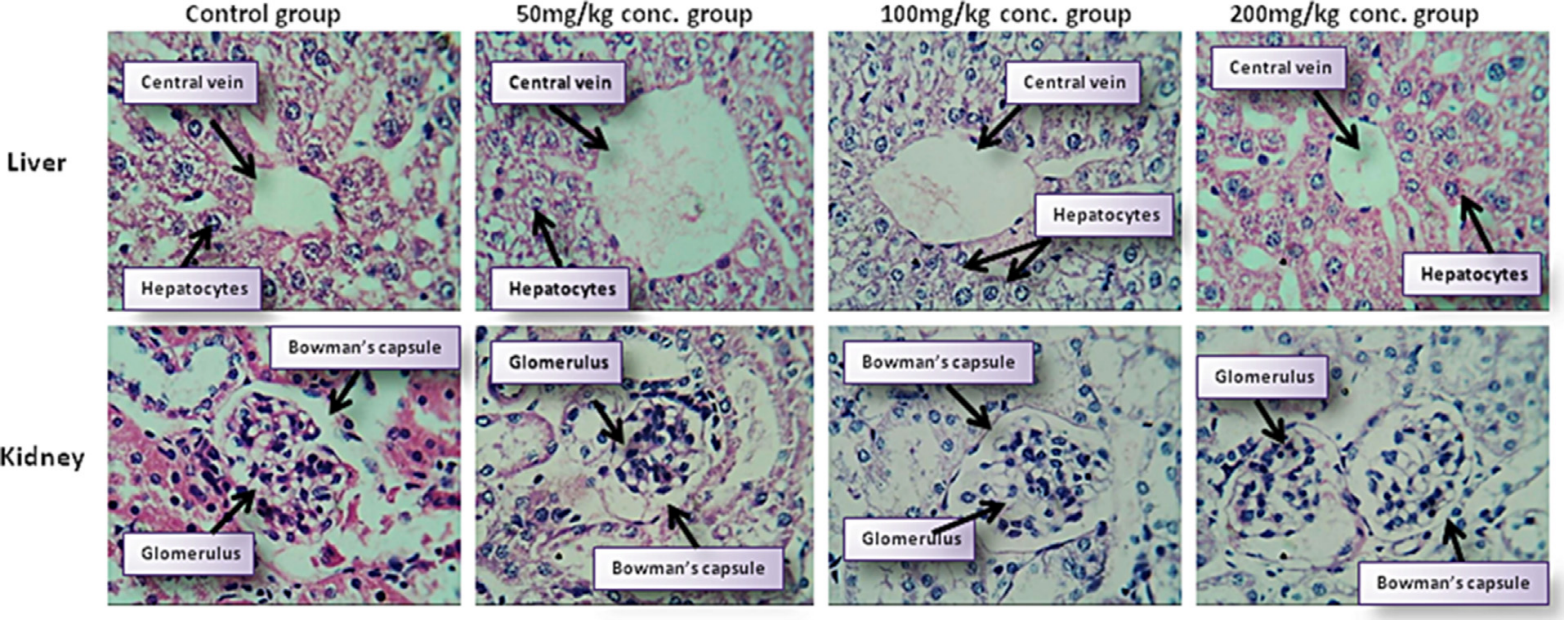
Histopathological examination of Liver and Kidney tissues of Swiss Albino mice at 40X from control and different treatment groups of subacute toxicity study.

Macroscopic examination of the organs of extract-treated mice did not show any color differences when compared to the control group. In comparison to the control group, macroscopic observation of the organs of extract treated animals revealed no color changes. Histopathology study of the liver and kidneys of both control and treated animals at the end of the trial exhibited no obvious alterations. When examined under a light microscope with several magnification powers, all of the organs from the extract-treated mice showed no changes in cell structure or adverse consequences. In both acute (Fig. 4) and subacute toxicity study groups (Fig. 5), the organization or coordination of cells in extract-treated organs was nearly similar to that of a control group.

### 3.9 In silico based screening of *Smilax china* to identify G6PD inhibitors

For identification of G6PD inhibitor compounds from *Smilax china* root extract, phytochemical analysis was performed via database searching and a list of literature-reported *Smilax china*-derived compounds was compiled. The PubChem database was used to obtain the structures of these phytochemicals (ligands) and molecular docking was used to screen them against G6PD binding sites. *Smilax china* derived 60 compounds were docked as for 2 binding sites of G6PD (PDB ID: 2BHL). Table 4 showed a relative examination of docking against two G6PD binding sites.

**Table 4:** Docking results of *Smilax china* derived compounds against two binding sites of G6PD.

Compound Name	G6P Binding Site	NADP^+^ Binding Site
	MolDock Score	MolDock Score
Scirpusin A	−139.018	−156.832
Smilachinin	−120.88	−148.215
Daucosterol	−117.76	−145.733
Bismilachinone	−119.112	−141.484
Kaempferol 3-O-beta-d-glucopyranosyl-7-O-alpha-l-rhamnopyranoside	−124.867	−141.099
Taxifolin-3-O-glycoside	−95.0748	−139.607
Isoquercetin	−104.886	−133.964
Astilbin	−103.472	−130.638
Rutin	−121.505	−130.408
Engeletin	−102.084	−127.796
Beta-sitosterol	−114.827	−124.55
Piceid	−116.512	−123.764
Isoengeletin	−84.8996	−122.698
Sieboldogenin	−115.57	−120.735
Kaempferol 7-O-α-Lranmnoside	−106.274	−119.37
Afzelin	−96.203	−118.075
Eicosanoic acid	−109.465	−117.935
8,11,14-Eicosatrienoic	−115.005	−117.822
5-O-caffeoylquinic	−106.939	−117.773
Telfairic acid	−118.535	−116.456
Quercetin-4-O-β-Dglucoside	−115.987	−115.808
Kaempferitrin	−116.978	−115.365
*cis*-vaccenic acid	−121.952	−115.166
Kaempferol-7-O-*b*- Dglucoside	−107.865	−114.759
9,12-Octadecadienoyl chloride, (Z,Z)	−108.702	−114.601
Oleic Acid	−121.526	−113.317
Vitexin	−108.212	−112.63
7-hexadecenoic acid methyl ester	−102.28	−111.059
Puerarin	−98.2772	−109.936
Sapogenin	−96.0286	−108.503
Lepidoside	−108.983	−107.402
Quercetin	−79.835	−106.269
Catechin	−93.4922	−105.482
Morin	−82.277	−104.474
Kaempferin	−85.168	−103.368
Oxyresveratrol	−88.4231	−103.231
Kaempferide	−87.615	−103.092
Kaempferol	−86.6424	−102.236
Pratensein	−90.3547	−101.029
Taxifolin	−88.0608	−100.961
Resveratrol	−93.9773	−100.028
Naringenin	−87.8881	−99.4347
Epicatechin	−81.1273	−97.6554
1,3,7-trimethyl-2,6-	−71.4812	−83.1421
Friedelin	−90.4595	−83.084
Cinchonin	−83.0311	−81.6776
delta-Cadinene	−66.2952	−81.0102
Caffeic acid	−77.8597	−79.1567
Cycloisolongifolene, 9,10-dihydro	−79.1437	−74.9617
Caffeine	−60.0553	−73.7473
Protocatechuic acid	−68.4948	−70.6354
Cedrol	−78.3807	−68.5484
Hydroxyflavan	−68.6167	−66.3426
2,5-dimethyl-2,4-Dihydroxy-3(2H)-furanon	−63.4784	−64.759
Terpineol	−61.6156	−60.992
Digitoxin	−43.3356	−58.0265
Pyrogallol	−52.2883	−55.7846
3-thujanol	−65.1518	−53.7363
1,5-anhydro-6- deoxyhexo-2,3-diulose	−48.9168	−52.8037
1,2,3-propanetriol	−48.5524	−44.3105

**Fig. 6:**
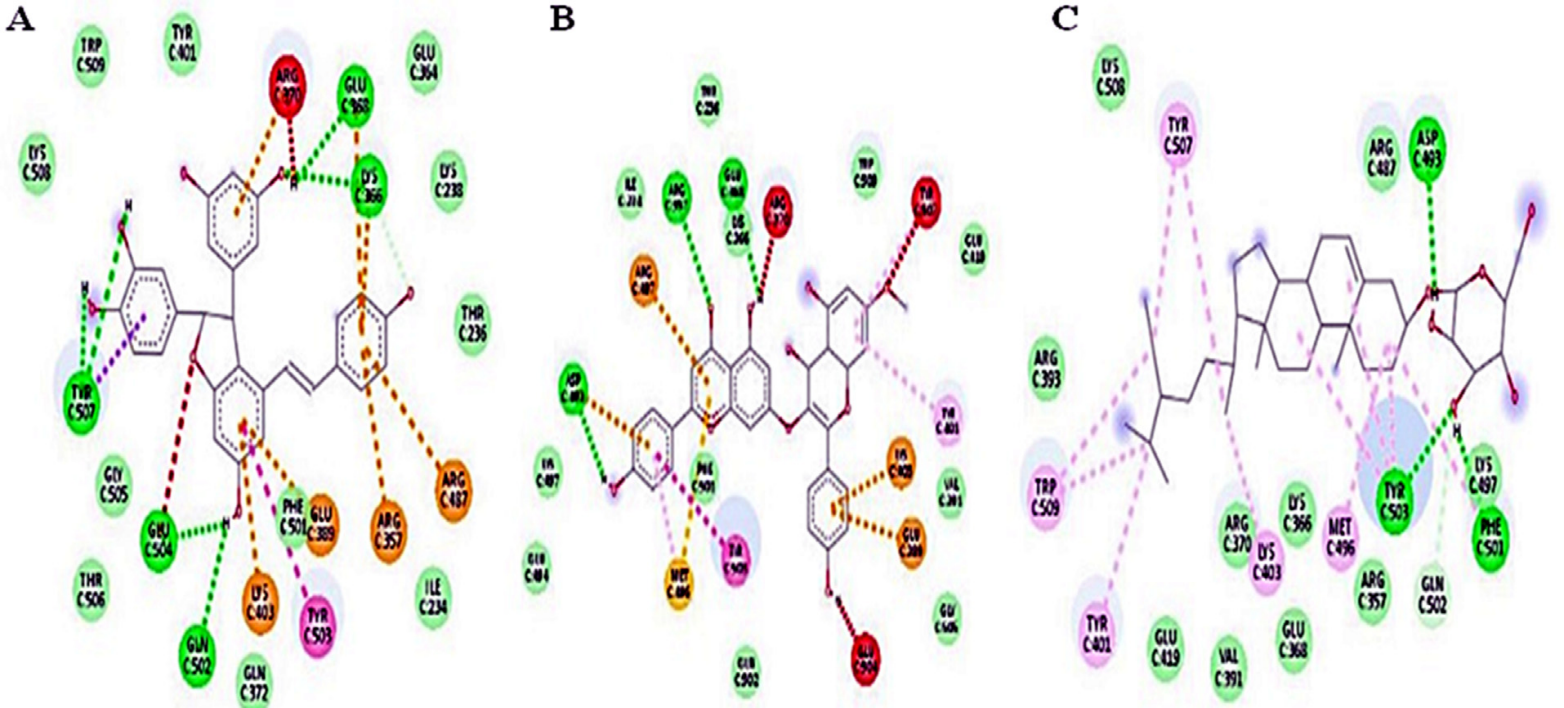
Docking complexes of the top three *Smilax china* compounds, (A) Scirpusin A, (B) Smilachinin and (C) Daucosterol within the NADP^+^ binding site of G6PD.

**Fig. 7:**
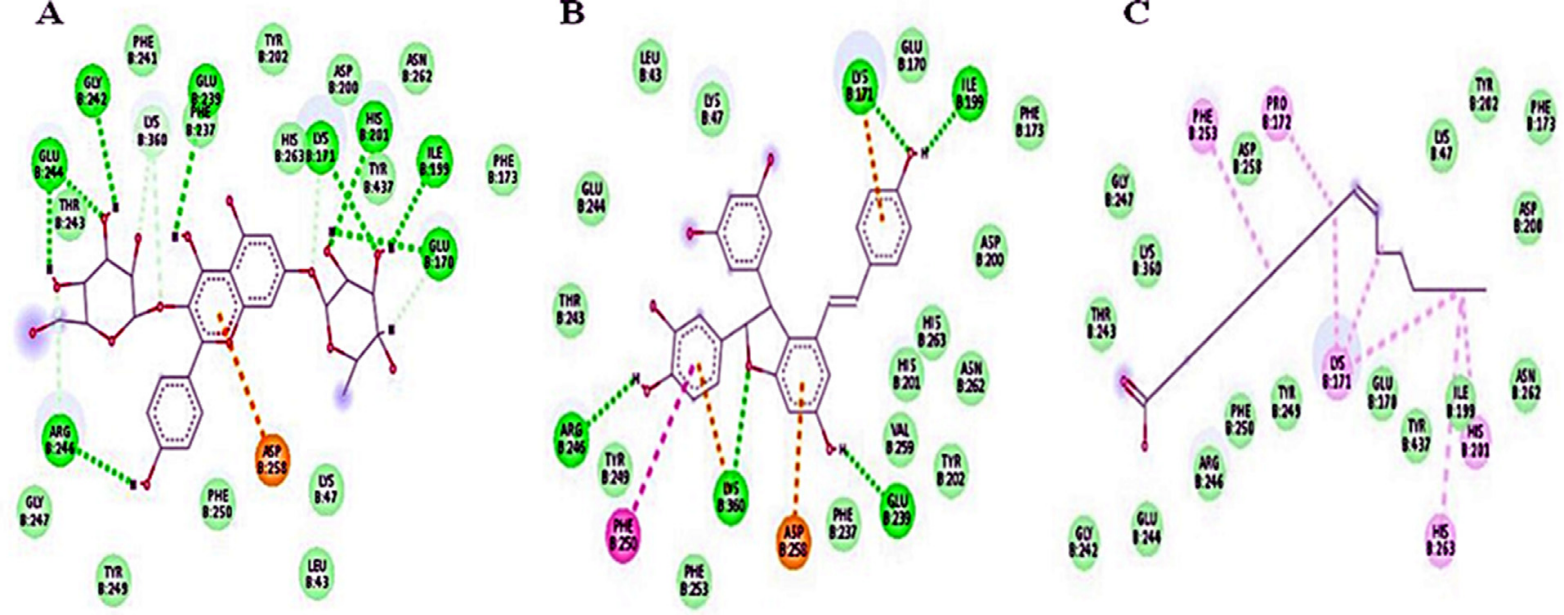
Docking complexes of the *Smilax china* compounds (A) Kaempferol 3-O-beta-d-glucopyranosyl-7-O-alpha-l-rhamnopyranoside; (B) Scirpusin A and (C) *cis*-vaccenic acid within the G6P binding site of G6PD.

In terms of binding affinities, 10 compounds are found to show good binding energies (MolDock Score of >125) to one or two binding sites. One out of ten compounds showed good binding attraction towards both binding sites of G6PD. Scirpusin A, Smilachinin and Daucosterol are the top three hits. By generating conventional hydrogen bonds and Van der Waals forces, these compounds have a good binding affinity with the target protein. The docking complexes of the top three *Smilax china* derived compounds against the NADP+ and G6P binding sites of G6PD are shown in Fig. 6 and Fig. 7.

## 4 Discussion

Glucose-6-phosphate dehydrogenase activity is more advanced in cancerous cells than in normal cells, and blocking G6PD in cancer cells causes diminished proliferation and greater apoptosis in vitro (Preuss et al., 2013). Taking into account that G6PD essentially contributes to cancer cell multiplication, metastasis and survival, the development of potent and particular G6PD inhibitors might open up new avenues for cancer therapy (Hu et al., 2015).

In this study after screening the plant extracts library, the root extract of *Smilax china* is identified with inhibitory activity against G6PD at a final dose of 4 µg/ml with an IC_50_ value of I.397 µg/ml. Our findings are comparable with the results of Mele et al., (2018), demonstrating that a natural molecule polydatin directly inhibits G6PD, resulting in redox imbalance leading to apoptosis, ER stress and cell cycle arrest. Another natural compound resveratrol isolated from the fruit of *Vitis vinifera* was found to decrease G6PD activity leading to anticancer activity in MCF-7, HeLa and HepG2 cell lines (Khan et al., 2020).

In agreement with this study, the anticancer potential of *Smilax chin*a (rhizome, leaf, bark) extracts has previously been reported against various tumor cell lines including A549 (Fu et al., 2017), HepG2 and MDA-MB-231 (Nho et al., 2015) and HeLa cells (Tettey et al., 2020). Molecular docking was used to screen *Smilax china*-derived compounds against G6PD to uncover novel natural scaffolds from *Smilax china* and to provide more opportunities for anti-cancer drug exploration. Several modulators of G6PD have been found by in silico screening as having the capacity to bind to the NADP+ and G6P binding sites of G6PD. One of the identified hits, Scirpusin A has been previously found to inhibit the advancement of colorectal cancer Her2/CT26 cells in mice by inducing apoptosis in the cells (Hong et al., 2017). Another hit compound daucosterol halts the advancement of the MCF-7 human breast cancer cells as well as the MGC803, BGC823, and AGS gastric cancer cells (Zhao et al., 2015), and promoted intrinsic apoptotic cell death in A549 cells (Rajavel et al., 2019). Another study reported that dacosterol inhibited prostate cancer growth in part by stimulating JNK signaling, which caused autophagic-dependent apoptosis (Gao et al., 2019). Comparing the results of our study with the previous findings suggests the anticancer potential of these compounds possibly because of G6PD Inhibition.

## 5 Conclusions

Plant extracts with the ability to suppress G6PD were screened using an enzymatic assay-based screening method. *Smilax china* root extract was identified as a possible inhibitor of G6PD as a result of this screening. Although *Smilax china* root extract has previously been claimed to have anti-cancer properties, the mechanism behind it is unknown. To the best of my knowledge, this is the first study that reveals the anti-cancer potential of *Smilax china* against HepG2 cells by G6PD suppression. Furthermore, the findings of acute and subacute toxicity studies in mice revealed that *Smilax china* root extract is nontoxic. As a result of in silico screening, numerous G6PD modulators such as Scirpusin A, Smilachinin, and Daucosterol were discovered from *Smilax china*. In future, it is strongly recommended to test these compounds for in vitro analysis to find their inhibitory potential against G6PD. Conclusively, the findings of this study present new insight into the probable mechanism of action of *Smilax china* root extract against hepatocellular carcinoma.

## Declaration of Competing Interest

The authors declare that they have no known competing financial interests or personal relationships that could have appeared to influence the work reported in this paper.
